# Efficient Secretion of Murine IL-2 From an Attenuated Strain of *Clostridium sporogenes*, a Novel Delivery Vehicle for Cancer Immunotherapy

**DOI:** 10.3389/fmicb.2021.669488

**Published:** 2021-06-08

**Authors:** Aleksandra M. Kubiak, Tom S. Bailey, Ludwig J. Dubois, Jan Theys, Philippe Lambin

**Affiliations:** ^1^The M-Lab, Department of Precision Medicine, GROW - School of Oncology, Maastricht University, Maastricht, Netherlands; ^2^Exomnis Biotech BV, Oxfordlaan, Maastricht, Netherlands

**Keywords:** streptolysin S, haemolysis, *Clostridium*, secretion, cytokine, spore, cancer

## Abstract

Despite a history dating back to the 1800s, using *Clostridium* bacteria to treat cancer has not advanced beyond the observation that they can colonise and partially destroy solid tumours. Progress has been hampered by their inability to eradicate the viable portion of tumours, and an instinctive anxiety around injecting patients with a bacterium whose close relatives cause tetanus and botulism. However, recent advances in techniques to genetically engineer *Clostridium* species gives cause to revisit this concept. This paper illustrates these developments through the attenuation of *C. sporogenes* to enhance its clinical safety, and through the expression and secretion of an immunotherapeutic. An 8.6 kb sequence, corresponding to a haemolysin operon, was deleted from the genome and replaced with a short non-coding sequence. The resultant phenotype of this strain, named *C. sporogenes*-NT, showed a reduction of haemolysis to levels similar to the probiotic strain, *C. butyricum* M588. Comparison to the parental strain showed no change in growth or sporulation. Following injection of tumour-bearing mice with purified spores of the attenuated strain, high levels of germination were detected in all tumours. Very low levels of spores and vegetative cells were detected in the spleen and lymph nodes. The new strain was transformed with four different murine IL-2-expressing plasmids, differentiated by promoter and signal peptide sequences. Biologically active mIL-2, recovered from the extracellular fraction of bacterial cultures, was shown to stimulate proliferation of T cells. With this investigation we propose a new, safer candidate for intratumoral delivery of cancer immunotherapeutics.

## Introduction

*Clostridium sporogenes* has been an important species in the field of experimental oncology for over 50 years, due to its ability to colonise solid tumours (Gericke and Engelbart, 1964; Heap et al., 2014). The hypoxic and necrotic environment of these tumours is ideal for the germination and growth of proteolytic *Clostridium* species, such as *C. sporogenes*. Intravenously injected spores germinate and proliferate exclusively in the hypoxic/necrotic environment of tumour xenografts, while vegetative cells are not detected in non-tumour-bearing control animals (Mowday et al., 2016). Injected spores are very well tolerated in rodent models, indicating low immunogenicity (Theys et al., 2001). While colonisation of tumours can lead to destruction of the necrotic portion of solid tumours, the potency of *C. sporogenes* alone appears to be inadequate for complete elimination of tumours. The absolute requirement of anoxic conditions, essential for initial colonization, may also explain why the proliferating *Clostridium* in the centre of a solid tumour are unable to attack the oxygenated fraction of the tumour, which constitutes the viable, proliferating portion of the cancer. A therapeutic agent, secreted by a recombinant *C. sporogenes* from the anoxic centre of a tumour, could overcome this limitation if it is able to diffuse to the living portion of the tumour and exert its effect.

Progress in understanding the biology of *Clostridium* species, and the concomitant development of genetic tools and genetic transformation, has enabled researchers to exploit this genus in the industrial and medical settings. *Clostridium* Directed Enzyme Prodrug Therapy (CDEPT) is an illustration of these developments (Mowday et al., 2021). CDEPT exploits the tumour-specific germination of an engineered *C. sporogenes*, which expresses a prodrug-converting enzyme from the bacterial chromosome to activate a systemically administered prodrug. Significant therapeutic effect was achieved in a mouse xenograft model (Heap et al., 2014).

*C. sporogenes* is also an important species for the food industry, where it is utilised as a surrogate for proteolytic *Clostridium botulinum* to test the efficacy of thermal processing (Brown et al., 2012). This sterilisation technique aims to destroy microbes, including spores, present in the food. Survival of *C. botulinum* is a concern due to its ability to germinate and produce the botulinum neurotoxin in anaerobic environments. At the nucleotide level, *C. sporogenes* shares 93.4% sequence identity with proteolytic *C. botulinum* (group 1), just short of the threshold for classing them as the same species (95%) (Weigand et al., 2015). The near identical phenotype without the risk of exposure to botulinum toxin makes this species an ideal surrogate. Inevitably, the significant similarity between these species has led to the misdiagnosis of pathogenic clostridia as *C. sporogenes* (Lindstrom et al., 1999). A pubmed search with the query “*Clostridium sporogenes*[title]” reveals five clinical reports in the last 30 years purporting *C. sporogenes* as the causative agent of infection. Validation of these cases is not possible due to use of inadequate methods or insufficient detail of diagnostics reported.

*C. sporogenes* is ubiquitous in the natural environment, and there is a growing weight of evidence to support the notion that this species is a beneficial member of the human gut microbiome. Studies in mice demonstrate its ability to produce a potent antioxidant and to modulate IgA-related immune cells via production of branched short chain fatty acids (SCFAs) (Wikoff et al., 2009; Dodd et al., 2017; Guo et al., 2019). Despite the good press, the presence of a nine gene cluster in *C. sporogenes* ATCC 15579 with high sequence similarity to the Streptolysin S (SLS) operon of *Streptococcus pyogenes* is a significant hurdle to its therapeutic use (Gonzalez et al., 2010).

We hypothesised that *C. sporogenes* NCIMB 10696 could be attenuated by deletion of the SLS operon without loss of the beneficial properties of the parental strain. The resultant strain would be safer for clinical and industrial use. To test this hypothesis, we deleted the SLS operon from this strain using CRISPR-Cas9, and conducted a comparative phenotype analysis with the parental strain. We have named this strain *C. sporogenes*-NT (non-toxic). In addition, we tested the new strain’s ability to express and secrete the murine cytokine, interleukin-2 (IL-2). This cytokine is the ideal candidate for intratumoral expression, due to its very high ED50 when given intravenously and the frequent occurrence of adverse events in patients (Buchbinder et al., 2019).

## Materials and Methods

### Bacterial Strains, Growth Conditions and Cell Lines

Details of all bacterial strains used in this study are listed in Supplementary Table 1. *C. sporogenes* NCIMB 10696 wild type strain was purchased from the NCIMB culture collection, received in a lyophilized state, and processed in accordance with NCIMB specifications. Two *Escherichia coli* strains (10-beta and S17-1) were used as the cloning and conjugative donor strains, respectively.

Growth of *C. sporogenes* strains was carried out under anaerobic conditions in an anaerobic cabinet (model MG1000 Mark II, Don Whitley Scientific Ltd.; 80% N_2_, 10% CO_2_, 10% H_2_) at 37°C. *C. sporogenes* was grown in a bovine-free version of TY media, labelled “Peptone Yeast Thioglycolate” (PYT), supplemented with D-cycloserine (250 μg/ml), and thiamphenicol (15 μg/ml) where appropriate. The CTLL-2 indicator cell line (93042610, ECACC) was cultivated according to the manufacturer’s instructions.

### Plasmid Construction and Isolation of *C. sporogenes*-NT Knockout Strain

Details of all vectors and oligonucleotides used in this study are listed in Supplementary Tables 1, 2. The pMTL82121 vector was digested with restriction enzymes *Not*I*/Asc*I and the ∼5 kb fragment was used as the backbone for the CRISPR-Cas9 gene editing plasmids (pPME-101-g1 and pPME-101-g2). The construction proceeded as previously reported (Heap et al., 2009; Ingle et al., 2019). The *cas9* gene was amplified from *S. pyogenes* genomic DNA using primers with upstream cloning sites (*Bsa*I/*Not*I). Expression of the *cas9* gene and the gRNA was achieved using the *thl* and *araE* gene promoters from *Clostridium acetobutylicum*, respectively, in a divergent arrangement. The 20-nt targeting sequences (guide 1 and guide 2) were designed using the CRISPR Guide RNA Design tool at the Benchling (2019) website. The gRNA DNA fragment was created using a conserved reverse primer (corresponding to the 3′ of the gRNA handle and terminator) and a forward primer containing the targeting sequence at the 5′ and the beginning of the gRNA handle at the 3′. Complementary sequences between these primers enabled the creation of a product in a template-free “primer dimer” reaction.

The SLS homologue was located in the *C. sporogenes* NCIMB 10696 genome by blastn alignment, using the equivalent genes in *C. sporogenes* ATCC 15579 as a query (Gonzalez et al., 2010). Sequences of approximately 700 bp long upstream and downstream of the operon were designated as left homology arm (LHA) and right homology arm (RHA), respectively. These were amplified from genomic DNA using Phusion^®^ high fidelity polymerase, according to the manufacturer’s protocol [M0531, New England Biolabs (NEB)]. A synthetic “bookmark” sequence (BM1) was placed between the homology arms. This was created by dividing the sequence between the LHA reverse and RHA forward primer tails with upstream *Bsa*I restriction sites.

Ligated plasmids were transformed by heat shock into *E. coli* 10-beta and plated on LB plates supplemented with chloramphenicol. Resultant colonies were PCR-screened for correct assembly and subsequent plasmid samples were confirmed by Sanger sequencing.

**FIGURE 1:**
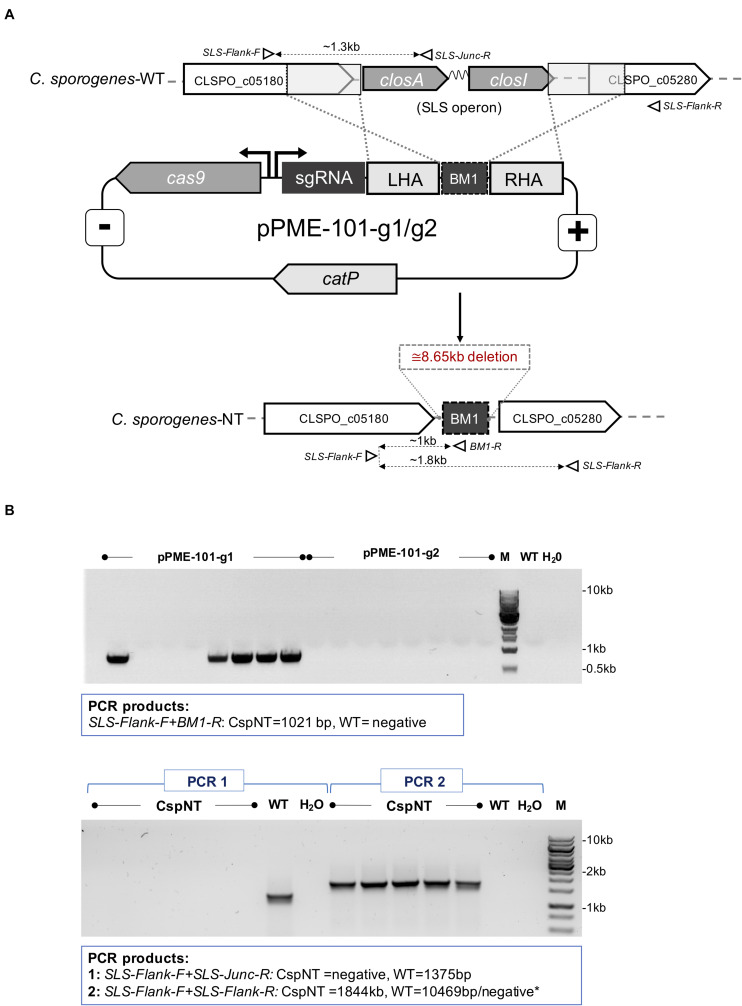
Schematic representation of CRISPR-Cas9 mediated integration and PCR analysis of SLS operon deletion in *C. sporogenes*-NT strain. **(A)** The genome editing vectors (pPME-101-g1/g2) containing spCas9, guide RNAs and an editing template for homologous recombination at the target chromosomal locus and integration of a BM1 “bookmark” sequence. Open triangles represent indicative alignment of screening primers as presented in Supplementary Table 2. **(B)** Gel electrophoresis showing a PCR screen of pPME-101-g1/g2 transconjugant colonies. Top image: Deletion of the operon detected in colonies subjected to a chromosome/bookmark-specific PCR reaction. Bottom image: Subsequent confirmation of SLS operon deletion in five selected clones. “M” denotes DNA marker, “dH_2_O” water template and “WT” *C. sporogenes* WT control.

Deletion of the haemolysin operon was achieved using the CRISPR-Cas9 genome editing tool (Ingle et al., 2019) and schematically illustrated in Figure 1A. Constructed knockout vectors (pPME-101-g1 and pPME-101-g2) were heat shock transformed into *E. coli* S17-1 for conjugation to *C. sporogenes*-WT. Conjugation was carried out as previously described (Heap et al., 2009). Transconjugants were screened by colony PCR using primers that flank the SLS-like operon genome locus. The CRISPR-Cas9 vector was removed from knockout mutants by subculturing in non-selective liquid cultures (24 h) followed by patch plating to selective and non-selective plates to observe loss of chloramphenicol resistance. Plasmid loss was further confirmed by PCR using primers specific for the plasmid-based *catP* gene (data not shown). Resultant positive clones were sent for sequencing. The haemolytic KO strain has been named *C. sporogenes*-NT.

### Construction of *C. sporogenes-*NT Secreting Murine IL2

To construct plasmids harbouring murine IL-2 secretory variants, the DNA sequence of *mIL-2* gene was manually codon optimised according to the known codon usage preference of *C. sporogenes* and based on the reported sequence (UniProtKB: P04351). The FLAG-tag sequence (DYKDDDDK) was added at the C-terminal end of the *mIL-2* gene. Five DNA fragments: FLAG-tagged recombinant mIL-2, and four *Clostridium*-derived promoter-signal sequences were commercially synthesized (IDT) with sites recognized by type IIS restriction enzymes (Supplementary Table 3).

To generate four mIL2 secretion variants, the promoter-signal sequence fragments and murine IL-2 were linearly ligated at *Bsa*I sites, followed by insertion to modified pMTL82121 *E. coli-Clostridia* shuttle plasmid (named pATB1C) at *Not*I and *Xho*I sites. A total of four secretion plasmids containing FLAG-tagged, codon optimised murine IL2 and combinations of promoter (P*fdx* or P*ptb*) and signal sequences (eglA or nprM3) were constructed (Supplementary Table 1). Plasmids were transferred to *C. sporogenes-*NT by conjugation from an *E. coli* donor as described previously (Heap et al., 2012). Resultant transconjugants were confirmed by colony PCR using primers specified in Supplementary Table 2 and by Sanger sequencing (Eurofins Genomics).

### Preparation of Pure Spore Suspensions

*C. sporogenes* strains were revived on agar plates and inoculated into 10 ml of growth media. The following day, the overnight cultures were inoculated into 500 ml of fresh media and cultivated under anaerobic conditions for six days. On day seven, cultures were removed from anaerobic cabinet and exposed to oxygen and room temperature for further 24 h. Next, spore-vegetative cell suspensions were centrifuged (10,000 *x g*, 20°C) for 30 min. Resultant pellets were washed two times with PBS and once with 70% (v/v) ethanol. The final pellet was resuspended in 10 ml PBS, heat treated at 80°C for 20 min to inactivate remaining viable vegetative cells and cooled down. The washed spore-debris suspensions were subjected to a density-gradient purification using Histodenz^TM^ (D2158, Sigma-Aldrich) according to the method of Setlow with minor modifications (Setlow, 2019; Supplementary Material). Spore purification was determined by counting phase bright spores and phase dark cells using a haemocytometer under a light microscope (minimum 200 bodies per purification). Spores still enclosed in the mother cell were counted as impurities.

### Phenotype Analysis of *C. sporogenes* Strains

#### Growth and Endospore Formation

The ability of *C. sporogenes*-NT spores to return to vegetative growth was evaluated by measuring the change in OD_600_ over a period of 24 h. Overnight cultures were inoculated into a fresh PYT media (1:100) and incubated at 37°C under anaerobic conditions.

A sporulation assay was carried out to measure and compare the ability of the *C. sporogenes* strains to form endospores. Growth media was inoculated with overnight cultures and incubated for 4 h. Following this incubation, fresh sterile broth was inoculated with the 4 h cultures (1:100) and incubated for 120 h. To determine spore titres, samples were taken every 24 h for five days and were heated at 80°C for 20 min to inactivate vegetative cells. Heat-treated samples were serially diluted and plated under anaerobic conditions. After 24 h incubation, colonies were counted, and CFU/ml was calculated to determine sporulation efficiency. A *spo0A* mutant, in which the master regulator of sporulation has been deleted, was used as a negative control for colony formation after heat treatment.

#### Measuring Haemolysis

*C. sporogenes* wild type and knockout strains were assayed for haemolysis by two methods. Plate assays were carried out using columbia sheep blood agar plates (PB0123, Oxoid) according to the ASM haemolysis protocol (Buxton, 2005). In addition, a liquid assay was conducted according to a published method (Totten et al., 1995). Briefly, 200 μl PBS-washed cultures were incubated in a 96-well plate with 20 μl washed whole sheep or horse blood for one hour. The plates were then centrifuged (200 *x g*, 10 min) and the supernatants transferred to a new sterile 96-well plate. Release of haemoglobin from lysed red blood cells (RBCs) was measured at OD_540_. Incubation with ultrapure water was used to determine complete lysis and with PBS to determine background lysis. In both assays, *C. butyricum* MIYAIRI 588 (CBM 588) and *S. pyogenes* were used as negative and positive haemolysin controls, respectively.

#### *In vivo* Colonisation Study

All animal experiments were performed in accordance with local institutional guidelines for animal welfare and were approved by the Animal Ethical Committee of the University of Maastricht (AVD1070020173367, Maastricht, Netherlands). Exponentially growing CT26 mouse colon carcinoma cells (*Mus musculus*, ATCC CRL-2638^TM^) syngeneic to the Balb/c mice were cultured in Roswell Park Memorial Institute (RPMI) (Lonza) supplemented with 10% fetal calf serum (FCS) in a humidified 5% CO_2_ chamber at 37°C. To induce tumours, approximately 8-week-old immunocompetent Balb/c OlaHsd mice (10) were subcutaneously injected with CT26 tumour cells (2 × 10^6^), resuspended in basement membrane matrigel (354234, BD Biosciences). When tumour volumes reached 200 mm^3^ (determined by measurement of three orthogonal axes with vernier calipers), treated animals (8) were given 1 × 10^6^ purified spores, administered intravenously in the lateral tail vein. Control animals (2) were injected with PBS. Seventy-two hours after spore administration, animals were sacrificed and tumours, spleens and lymph nodes were excised.

#### *Ex vivo* Sample Processing

Blood was collected in 10% heparin sodium (5000 I.U./ml, Leo Laboratories Ltd.) and plasma was separated by centrifugation (1600 *x g*, 4°C, 5 min). Red blood cells were removed from the remaining cell suspension using a RBC lysis buffer (00-4333-57, eBioscience). Single cell suspensions of lymph nodes, spleens and tumours were obtained using a gentleMACS dissociator (130-093-237, Miltenyi Biotec B.V.) and filtered through a 70 μm-pore cell strainer (542070, Greiner Bio-one).

The presence of spores and vegetative cells of *C. sporogenes*-NT in each tissue was determined by dilution plating on selective media. For total colony forming unit (CFU) counts, single cell suspensions were serially diluted in pre-reduced PBS to 10^–7^. For each dilution, three 20 μl spots were dispensed on to pre-reduced agar plates. The plates were incubated for 24 h and then observed for single colony growth and counted. Dilution plating was also conducted on heat-treated samples (80°C, 20 min) to quantify spores present. Vegetative cells were quantified by deducting heat-treated counts from non-heat-treated counts. Counts of spores and vegetative cells in tumours were divided by equivalent counts in the same weight of other tissues to give a ratio of the localisation in each tissue. Using bookmark-specific primers, colony PCR was conducted to confirm that the observed CFUs were the *C. sporogenes*-NT.

#### Detection of Recombinant mIL-2

The secretion of mIL-2 protein in *C. sporogenes*-NT-XmIL2F variants was confirmed by Western blotting in DOC-TCA precipitated fractions of culture supernatants using anti-FLAG antibody according to the related literature and manufacturer’s recommendations (Schwarz et al., 2007).

The levels of mIL-2 secreted from *C. sporogenes*-NT in 7 h culture supernatants were determined by cytokine specific ELISA assay (BMS601, Invitrogen) in accordance with the manufacturer’s instructions. The results were recorded in a microplate reader (BMG Labtech SPECTROstar Omega) and calculated based on recombinant mIL2 standard.

The biological activity of the secreted cytokine was determined in a lymphocyte proliferation assay using the CTLL-2 T cell line. Seven-hour subcultures of mIL2-expressing *C. sporogenes*-NT were centrifuged (10,000 *x g*, 10 min), filtered (0.45 μm syringe filter) and the sterile supernatants retained. Supernatant-stimulated CTLL-2 proliferation was measured using the MTT assay according to published methods (Soman et al., 2009) and described in detail in Supplementary Materials. The standard curve was prepared using purified recombinant mIL2 (212-12, Peprotech). Absorbance was recorded in a microplate reader (BMG Labtech SPECTROstar Omega).

#### Biological Replicates and Statistical Analyses

All data presented in this manuscript represent the results of at least three independent experiments. Statistical evaluations were performed with GraphPad Prism 8 software (San Diego, CA, United States). For the ELISA and lymphocyte proliferation assay, data was analysed using unpaired *t*-test to compare *C. sporogenes*-WT with each mIL2 variant. To compare haemolysis in *C. sporogenes*-WT and *C. sporogenes*-NT, data was analysed using two-way ANOVA with Dunnett’s multiple comparison test. Values of *p* < 0.05, *p* < 0.01, *p* < 0.001 were considered significant (^∗^), highly significant (^∗∗^), or extremely significant (^∗∗∗^) respectively. Data represent means ± standard deviation (SD).

## Results

### Deletion of the SLS Homologue Causes a Significant Reduction in Haemolysis

The identification of streptolysin S in the genome of *C. sporogenes* NCIMB 10696 highlighted a potential risk of using this species as an intratumoral delivery vehicle. We sought to determine whether deletion of this virulence factor affected this strain’s haemolysis capability. Two CRISPR-Cas9 vectors were created with the aim of deleting the SLS operon, distinguished by the presence of two different targeting sequences, guide 1 (g1) and guide 2 (g2) (Figure 1A). Following conjugation, eight colonies for each vector were screened by PCR for chromosomal recombination. For the vector with g1, deletion was detected in five out eight transconjugants (Figure 1B). Deletion of the operon at the SLS locus was confirmed by Sanger sequencing. For transconjugants harbouring the vector with g2, the WT sequence was detected at the SLS locus, indicating that chromosomal recombination had not occurred.

**FIGURE 2:**
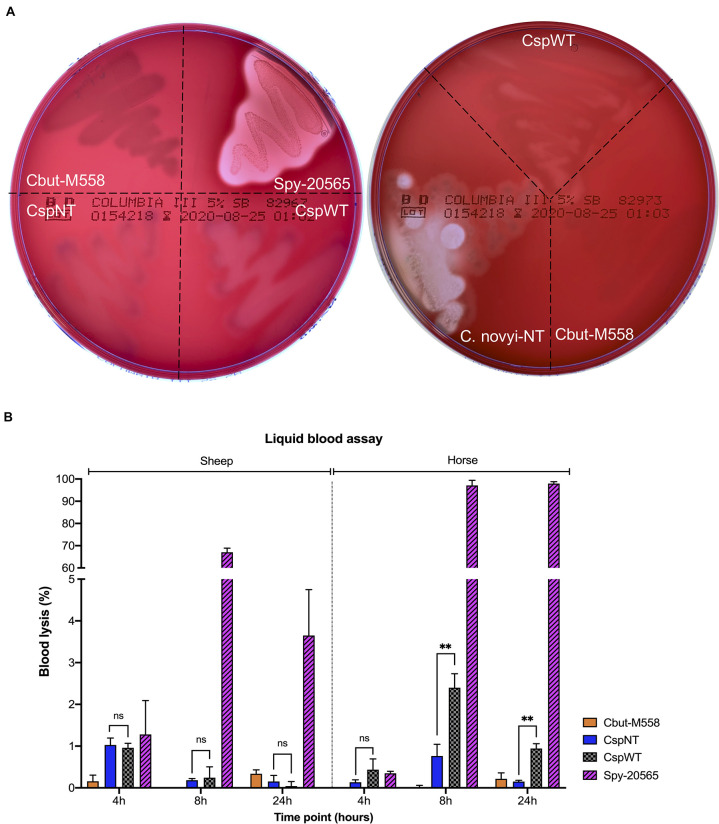
Analysis of haemolytic activity. **(A)** Five strains were streaked on two Columbia Blood Agar plates (5% sheep blood BD Bioscience) and incubated under anaerobic conditions for 48 h. The strains were: Cbut-M558: *C. butyricum* MIYAIRI 585 (negative control), Csp-20565: *S. pyogenes* DSM 20565 (positive control), CspNT: *C. sporogenes*-NT, CspWT: *C. sporogenes*-WT and *C. novyi*-NT. **(B)** Quantitative analysis of haemolysis in two liquid blood assays (using sheep and horse blood). Samples from liquid cultures were assayed at 4, 8, and 24 h. Haemolysis is presented as a percentage of total lysis (blood incubated with ultrapure water). Assayed strains: Cbut-M558: *C. butyricum* MIYAIRI 588; CspNT: *C. sporogenes*-NT; CspWT: *C. sporogenes*-WT; Spy-20565: *S. pyogenes* DSM 20565.

Neither CspWT nor CspNT strains of *C. sporogenes* displayed strong beta-haemolysis at 24 or 48 h after streaking on blood plates, in contrast to the positive control, *S. pyogenes* (Figure 2A). A validated liquid assay with horse and sheep blood was employed to quantify haemolysis (Figure 2B). In our experiments, horse blood was more sensitive to haemolysis than that of sheep. Significant differences could not be detected in sheep blood. In horse blood, differences between 4-h cultures of each strain were difficult to distinguish, and differences were not statistically significant. At 8 h, the difference in haemolysis between CspWT and CspNT was most pronounced (CspWT 2.5% ± 0.27 SD; CspNT 0.76% ± 0.23 SD). At this time point CspNT haemolysis was not higher than that of the non-haemolytic control, Cbut-M588 (*p* = 0.371). At 24 h, CspWT haemolysis remained elevated (0.94% ± 0.09 SD) while for CspNT it was comparable to Cbut-M588. At both 8 and 24 h, the difference between CspWT and CspNT was statistically significant (*p* < 0.05).

Following the announcement of a first in human (FIH) study utilising *C. novyi*-NT, we sought to benchmark *C. sporogenes*-NT to compare haemolysis. Growth of *C. novyi*-NT and *C. sporogenes*-NT on sheep blood was observed. In contrast to *C. sporogenes*-NT, *C. novyi*-NT caused significant beta-haemolysis (Figure 2A). Sequence similarity to the SLS operon could not be detected in the *C. novyi*-NT genome when using protein (tblastn) or DNA (blastn) as queries.

**FIGURE 3:**
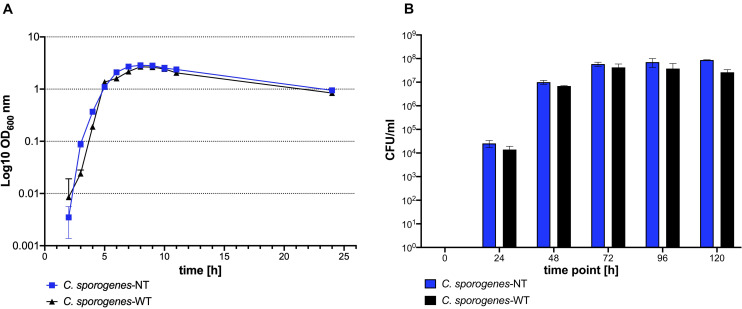
Growth and development of heat resistant colony forming units of *C. sporogenes*-NT (blue) and *C. sporogenes*-WT (black) strains. **(A)** Growth of *Clostridium* strains was measured as a direct increase in absorbance at 600 nm throughout the course of 24 h bacterial incubation in bovine free medium (PYT). The symbols represent the average of three independent experiments, and error bars indicate the standard errors of the means. **(B)** Heat-treated bacterial samples (80°C, 20 min) were plated in serial dilution on PYT agar plates and enumerated following 24 h incubation. Bars represent the number of CFU (colony forming unit) per ml of *Clostridium* culture. The data represent the average of three independent experiments and error bars indicate the standard error of the mean. The sporulation-deficient *Clostridium sporogenes-*NTΔ*spo0A* mutant was used as a negative control to rule out experimental error. The detection limit for colony counts was 50 CFU/ml.

The ability to form spores is a characteristic feature of *C. sporogenes*. We sought to determine whether *C. sporogenes*-NT displayed the same growth and sporulation phenotype as that of the parental strain. The new *C. sporogenes*-NT strain grew and formed spores at the same rate and to a comparable final titre as the wild type (Figures 3A and 3B). Our data demonstrates that deletion of the SLS operon has no significant impact on growth and germination efficiency over a 24 h period or sporulation over 120 h.

### *C. sporogenes-*NT Colonises Tumours in Experimental Animals

**FIGURE 4:**
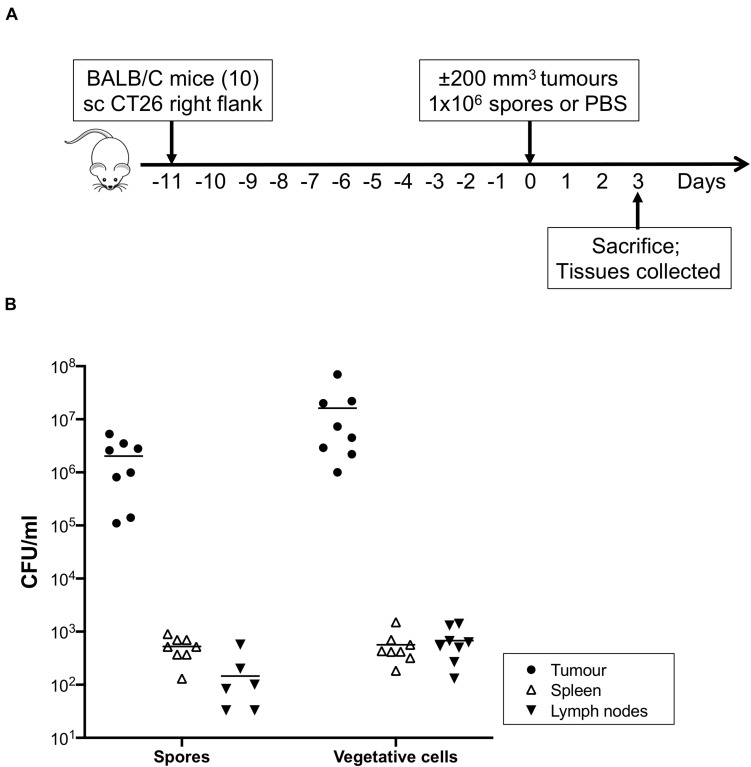
*In vivo* colonisation study and recovery of bacteria from mouse tissues. **(A)** Immunocompetent Balb/c mice (ten) were subcutaneously injected with CT26 tumour cells (2 × 10^6^). When tumour volumes reached approximately 200 mm^3^ eight treated animals were given 1 × 10^6^ purified *C. sporogenes*-NT spores. Two control animals were injected with PBS. 72 h after administration of spores, animals were sacrificed and tumours, spleens and lymph nodes were excised and subjected to cell counts. **(B)** The presence of spores and vegetative cells of *C. sporogenes*-NT in each tissue (tumour, black circles; spleen, open triangles; lymph nodes, inverted black triangles) was determined by dilution plating on selective media and expressed as colony forming unit (CFU).

The ability of *C. sporogenes* NCIMB 10696 to colonise solid tumours has been demonstrated previously (Heap et al., 2014; Mowday et al., 2021). We sought to determine whether deletion of the SLS operon has an effect on this ability. The CT26 murine colon carcinoma cell line is highly immunogenic. Cells were implanted subcutaneously to allow easy measurement of growing tumours. Tumours were formed in all ten mice injected with CT26 cells. Tumour-bearing animals were sacrificed 72 h after systemic administration of *C. sporogenes*-NT spores (eight) or PBS (two). An overview of the *in vivo* experiment is presented in Figure 4A.

The presence of spores and vegetative cells in tumours, spleens, lymph nodes and blood was determined (Figure 4B and Supplementary Table 4). Neither spores nor vegetative cells were detected in any tissues from control animals that were injected with PBS instead of spores, or in the blood of spore injected animals. In the tumours of spore-injected animals, spore counts ranged from 1.1 × 10^5^ – 1.7 × 10^6^ per ml. Vegetative cell counts were higher at 1 × 10^6^ – 2.2 × 10^7^ per ml. In the spleens of spore-injected animals, average counts of bacteria were 4,000 and 26,000 times lower than in the equivalent weight of tumour tissue, respectively. For lymph nodes, spores and vegetative cells were 62,000 and 81,000 times lower, respectively. Ratios were determined as outlined in the methods section. For all excised tissues, plate colonies were confirmed to be *C. sporogenes*-NT by PCR using bookmark-specific primers (Supplementary Table 2, data not shown).

### *C. sporogenes-*NT Secretes Biologically Active Murine IL-2 Cytokine

**FIGURE 5:**
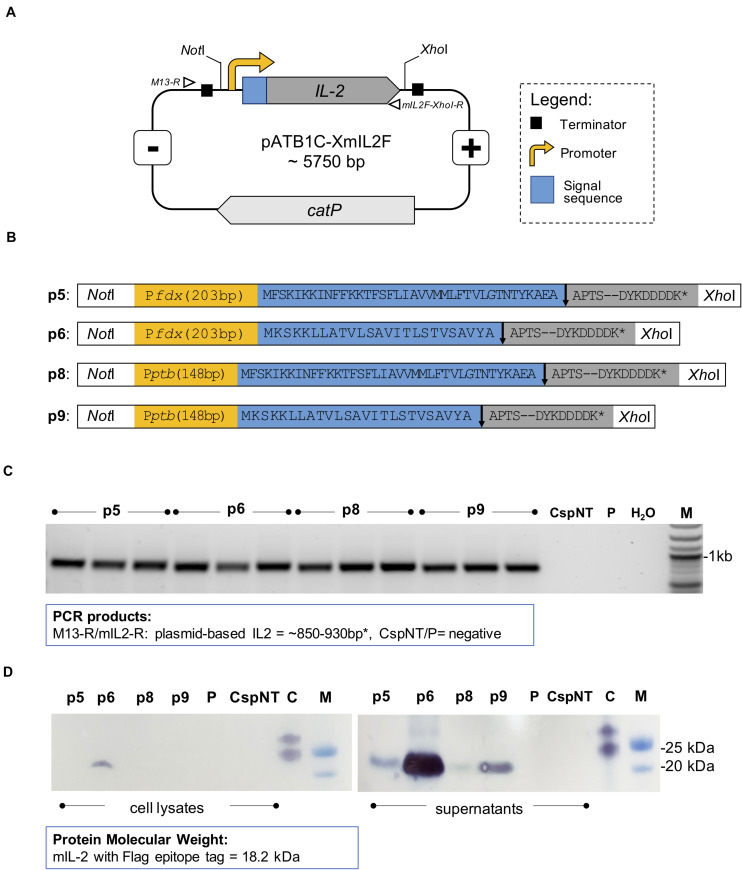
Design and confirmation of *C. sporogenes*-NT-XmIL2F variants. **(A)** Schematic illustration of expression vector pATB1C-XmIL2F. The codon optimised and FLAG-tagged murine *IL-2* gene was ligated at the *Bsa*I site with relevant promoter and signal sequence variants (Supplementary Table 3) and inserted into pMTL82121 vector at the *Not*I/*Xho*I sites. Open triangles indicate alignment of screening primers: M13-R and mIL2F-*Xho*I-R. **(B)** Four mIL2 expression modules consist of P*fdx* or P*ptb* promoter (yellow box) followed by eglA or nprM3 signal sequence (blue box). The FLAG-tagged murine *IL-2* gene (grey box) was ligated downstream of a relevant signal sequence. The arrow indicates the signal sequence cleavage site. **(C)** Colony PCR screening of *C. sporogenes*-NT transconjugants harbouring four variants of plasmid-based *mIL-2* gene. For each variant a total of three independent clones have been obtained. Plasmid-based mIL-2 strains were denoted p5, p6, p8 and p9 (Supplementary Table 1), “CspNT” denotes *C. sporogenes*-NT control, “P” denotes *C. sporogenes*-NT-pATB1C (empty vector), water (dH_2_0), M: DNA marker. **(D)** Visualisation of Western immunoblot analysis performed on *C. sporogenes*-NT-XmIL2F cell lysates and supernatant fractions following the incubation with FLAG-tag antibody. “C” denotes FLAG-tag positive control, “M” denotes protein marker. FLAG-tagged murine IL-2 protein has been visualised on the nitrocellulose membranes with TMB-Blotting 1-Step Solution and corresponds to the size of 18.2 kDa.

We tested the ability of *C. sporogenes*-NT to express and secrete a heterologous protein, exemplified using mIL-2. This cytokine has a short half-life and requires intravenous administration at high doses for clinical efficacy, resulting in adverse events in a high proportion of patients. Four pATB1C-XmIL2 plasmids with different promoter-signal sequence combinations were conjugated into *C. sporogenes*-NT and verified by colony PCR (Figures 5A–C) and Sanger sequencing. The impact of these variants on expression and secretion of mIL-2 was assessed. Testing multiple expression variants increased the chance of cloning a functional mIL-2 secreting strain without mutation or growth inhibiting host toxicity.

Western blot analysis was used to determine the presence of FLAG-tagged protein in the intra- and extra-cellular fractions of recombinant *C. sporogenes*-NT cultures. mIL-2 was detected in the supernatants of all *C. sporogenes*-NT variants and was absent in the cellular fraction of three out of four variants, indicating effective and complete secretion (Figure 5D). A small quantity of tagged protein was detected in the cellular fraction of variant p6. These results indicate that nrpM3 is an efficient secretion peptide. For both nprM3 and eglA signal peptides, variants utilising the P*fdx* promoter showed the highest expression levels.

**FIGURE 6:**
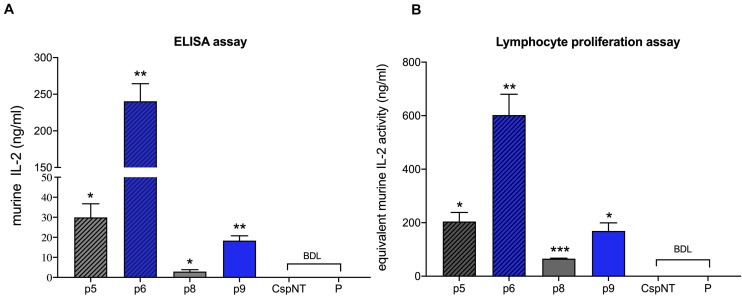
Validation of *C. sporogenes*-NT variants harbouring plasmid-based mIL-2 in the quantitative and functional assays. **(A)** Results of commercial ELISA test indicating the quantities of mIL-2 cytokine present in the supernatants of four *C. sporogenes*-NT-XmIL2F variants following 7-h growth in PYT media. **(B)** Results of colorimetric MTT functional assay following the incubation of CTLL-2 T-cells in the presence of 7-h culture supernatants of *C. sporogenes*-NT-XmIL2F variants. Recombinant murine IL2 was used to prepare mIL-2 standard curve. BDL, below detectable levels. Plasmid-based mIL-2 strains were denoted p5, p6, p8, and p9 (Supplementary Table 1), “CspNT” denotes *C. sporogenes*-NT control, “P” denotes *C. sporogenes*-NT-pATB1C (empty vector).

Secreted mIL-2 levels in 7-h cultures were quantified in an mIL-2 ELISA. Maximum mIL-2 levels were detected in variant p6 (240 ng/ml, *C. sporogenes*-NT-p6mIL2F), while the lowest levels were detected in variant p8 (2.9 ng/ml, *C. sporogenes*-NT-p5mIL2F) (Figure 6A). Next, we sought to confirm that the secreted product was biologically active. The murine cytotoxic T cell line CTLL-2 is dependent on pro-inflammatory cytokines for viability. By stimulating the growth of these cells in the presence of IL-2 standards or bacterial culture supernatant, the biological activity of secreted mIL-2 can be quantified. Supernatants from late-exponential (7 h) cultures of wild-type and mIL-2 expressing *C. sporogenes*-NT, were applied to washed CTLL-2 cells and incubated for 48 h. The MTT assay was used to quantify T cell proliferation. The final spectrophotometric detection confirmed that mIL-2 levels of 602 ng/ml (3010 U/ml) were measured for “p6”, the most potent recombinant *Clostridium* variant (*C. sporogenes*-NT-p6mIL2F), dropping to 65.7 ng/ml (328 U/ml) in variant “p8” (Figure 6B). These results indicate that mIL-2 expressed by recombinant *C. sporogenes*-NT is efficiently secreted and folds into a native conformation. All results confirmed that haemolysin-free *C. sporogenes*-NT strain is a suitable vehicle for the secretion of proteins, such as mIL-2 cytokine.

## Discussion

Haemolysis is a defining feature of many bacterial infections, including necrotising enterocolitis (*Clostridium perfringens*), haemolytic–uremic syndrome (*Escherichia coli*) and listeriosis (*Listeria monocytogenes*). Clinical laboratories routinely determine haemolysis by streaking patient specimens on blood agar plates. A strain is considered beta-haemolytic (displays “true haemolysis”) if it produces a clearing of the red pigment conferred by the blood (Madigan, 2012).

Publication of the genome sequences of *C. sporogenes* ATCC 15579 and a closely related Group 1 *C. botulinum* revealed an SLS homologue, which confers a haemolytic phenotype in *S. pyogenes* (Sebaihia et al., 2007). A subsequent study of this homologue concluded that the genes were functionally equivalent to those of *S. pyogenes* (Gonzalez et al., 2010). This was demonstrated by the independent deletion of four *S. pyogenes* M1 SLS genes (*sagA-D)* followed by complementation with the equivalent genes from *C. botulinum*. Complementation of *sagA* and *sagD* strains restored WT levels of haemolysis, while *sagB* complementation failed to restore haemolysis and *sagC* partially restored haemolysis. In contrast to our own results for *C. sporogenes* NCIMB 10696, this paper reports that *C. sporogenes* ATCC 15579 exhibits strong beta-haemolysis in blood plate assays. This suggests there is considerable variation in haemolysis between strains of *C. sporogenes*.

We studied the effect of the SLS homologue in *C. sporogenes* NCIMB 10696. An 8.6 kb region of the chromosome, corresponding to the SLS operon, was deleted. To accurately quantify haemolysis, we developed a liquid assay that proved considerably more sensitive than the conventional blood plate assay. This method enabled us to detect and quantify the effect of the SLS gene products, and the reduction of haemolysis following operon deletion. This decrease was statistically significant in horse but not in sheep blood. For blood agar cultures in clinical laboratories, sheep blood is commonly used in North America, while horse blood is more common in Europe. We were unable to find relevant studies in the literature that compare haemolysis in the blood of these two animals. Both sources are considered reliable for the detection of haemolysis (Jorgensen et al., 2015). Growth and sporulation were not affected by deletion of the SLS operon.

The ability of *Clostridium* species, including wild type *C. sporogenes* NCIMB 10696, to colonise hypoxic/necrotic tumours is well documented (Zhou et al., 2018). The most advanced example of this is a recently reported first-in-human (FIH) study using *C. novyi*-NT for treatment of treatment-refractory solid tumours (Janku et al., 2020). *C. novyi*-NT differs from its wild-type parental strain due to the absence of the alpha toxin. In preclinical studies it was determined that intratumoral injection was the best method of administration, due to dose-limiting toxicities observed following intravenous administration. Following intratumoural injection with *C. novyi*-NT, elevated cytokines as well as local and systemic tumour antigen-specific T cell responses were observed in patients that showed signs of germination. This response was not seen when signs of germination were absent. Three of the 24 patients experienced dose-limiting toxicities, including sepsis and gangrene. The results of the *C. novyi*-NT FIH study have demonstrated the feasibility of intratumoural *Clostridium* treatment and has justified further clinical studies (NCT03435952). Intravenous delivery of *C. sporogenes* secreting clinically approved immunotherapeutics is conceptually different. This method does not rely on the innate tumorolytic ability of the bacteria, rather it is a means to deliver therapeutics. In addition, the less invasive nature of the treatment could be advantageous.

We sought to determine the impact of deleting the SLS-like operon on the ability of *C. sporogenes* to colonise hypoxic/necrotic tumours in mice. In animals injected with *C. sporogenes*-NT spores, very high levels of spores and vegetative cells were detected in tumours compared to other healthy tissues (≥ 4000-fold). On average, 90% of the bacteria existed in the metabolically active vegetative form, indicating that the tumour environment promoted germination of the injected spores. Spores and vegetative cells were detected at very low levels in the spleen and lymph nodes. Neither of these tissue types are anoxic and, therefore, are unlikely to support growth of obligately anaerobic bacteria. Detection of spores and vegetative cells in these tissues may be due to accumulation from the initial injection or due to leakage from the tumour. These organs filter the blood and lymphatic system, respectively, so they are likely to concentrate any foreign bacteria present in these systems. Our results are consistent with a previous study of *Clostridium* localisation following intravenous administration (Lambin et al., 1998; Diaz et al., 2005).

*C. sporogenes*-NT could be exploited to increase the concentration of an immunotherapeutic at the tumour site without exposing healthy tissues, avoiding the adverse effects caused by systemic delivery. While commercial production of recombinant proteins has become commonplace, adapting these principles for therapeutic delivery raises a number of challenges. The therapeutic must be expressed and secreted from the bacteria before folding correctly to form the functional product. In addition, it must be delivered at levels at or above the effective dose, a function of expression, secretion, protein degradation and proliferation of the bacteria.

Interleukin-2 was one of the earliest immunotherapy treatments, but the significant disadvantages associated with systemic IL-2 treatment have led to more sophisticated versions, capable of targeting the cancer (Rekers et al., 2018; Lieverse et al., 2020). Approved for treatment of renal cell carcinoma and metastatic melanoma, IL-2 is a potent T cell activator (Amin and White, 2014; Payne et al., 2014). Despite preferentially inducing the expansion of immune-suppressing regulatory T cells (Tregs), IL-2 can produce significant clinical responses when administered systemically at high doses (600,000 IU/kg every 8 h) (McDermott et al., 2005). However, objective clinical response is only seen in 15-20% of patients, and immune-related adverse effects (irAEs) are a significant issue that can be treatment limiting (Buchbinder et al., 2019). Attempts to reduce toxicity by using lower doses of IL-2 results in a significant drop in therapeutic effect, due to the dominant effect of Tregs (Ahmadzadeh and Rosenberg, 2006). The high dose requirement and the risks of systemic toxicity make IL-2 an excellent test case for an intratumoral delivery system.

A panel of IL-2 expressing *C. sporogenes*-NT strains were created using different combinations of native promoters (two) and secretion peptide sequences (two). Expression of recombinant proteins using a single strong promoter carries a risk of failure, due to the potential toxicity to the host bacteria. This can manifest as growth inhibition and the emergence of more competitive mutant variants. Toxicity has been reported for recombinant expression of IL-2 (Mahmoudi Azar et al., 2013). To mitigate this risk, the mIL-2 coding sequence was cloned with two promoters, representing high and low expression. In our own experience, P*fdx* is consistantly strong in any genetic context, while P*ptb* produces significantly lower expression. This is in agreement with other studies (Brunt et al., 2014; Pyne et al., 2014). Secretion from Gram-positive bacteria can be achieved by addition of a secretion peptide to the N terminus of the protein, encoded upstream of the coding sequence. Signal peptides can be functionally interchangeable between species, however the efficiency of protein secretion is strongly determined by these leader sequences. In the context of mIL-2, the native nprM3 precursor greatly improved the efficiency of secretion over that of eglA from C*lostridium saccharolyticum*. High-level expression combined with inefficient secretion can lead to recombinant product accumulation as inclusion bodies, and corresponding host toxicity (Horga et al., 2018; Li and Rinas, 2020). The performance of signal peptides is coding sequence dependent, and predicting the secretion efficiency based on peptide sequence is not possible (Freudl, 2018). Identifying a range of secretion peptides that perform well in different contexts will facilitate future recombinant protein secretion. In this study, peak mIL-2 production was achieved in strains that utilised the P*fdx*-nprM3 promoter/secretion peptide combination. In this construct, a small quantity of tagged protein was detected in the cellular fraction of cultures. This could be a result of contamination of the cell pellet by residual supernatant prior to protein precipitation. Another possibility is that the rate of gene expression with the P*fdx* promoter exceeds the rate of secretion, leading to an accumulation of mIL-2 in the cytoplasm. In a T cell proliferation assay, culture supernatants of this strain showed biological activity equivalent to 602 ng/ml (3010 U/ml) while ELISA experiments indicated 240 ng/ml (1200 U/ml). The highly proteolytic nature of *C. sporogenes* is likely to reduce the level of secreted mIL-2 measured in laboratory batch cultures. *In vivo* experiments will provide an insight into the different dynamics of protein production and degradation inside a solid tumour. Prior to this research, secretion of recombinant rat IL-2 was demonstrated in *C. acetobutylicum*, a saccharolytic species from the same *Clostridium* cluster I. This study reported production of 800 ng/mL active cytokine, although this was measured at a significantly higher culture density (Barbé et al., 2005).

This paper reports a safer version of the well-studied cancer delivery vector, *C. sporogenes*, by reduction of toxicity through chromosomal gene editing. Our experiments revealed that *C. sporogenes* NCIMB 10696 was only weakly haemolytic, but the irrefutable presence of the streptolysin S homologue in the genome sequence could undermine confidence of regulatory bodies and patients. Deletion of a significant region of the genome (approx. 8.6kb) did not influence the attenuated strain’s ability to colonise solid tumours.

The new strain was transformed with mIL-2 expression vectors. Using a native gene promoter, significant quantities of mIL-2 were detected in the extracellular fraction of cultures. To improve the performance of this approach, the next step is to optimise recombinant gene expression and secretion, as well as expand the repertoire of therapeutic agents. Further optimisation will minimise the metabolic burden and potential toxic effect on the host bacteria while maximising dose and, by extension, therapeutic effect. In addition, chromosomal integration of optimised expression cassettes will be essential to ensure genetic stability and to eliminate the risk of horizontal gene transfer. Genetically engineered *C. sporogenes*-NT poses an elegant and highly adaptable solution to the challenge of precise delivery of anti-cancer agents.

## Data Availability Statement

The original contributions presented in the study are included in the article/Supplementary Material, further inquiries can be directed to the corresponding author/s.

## Ethics Statement

The animal study was reviewed and approved by Dier Experimenten Commissie (DEC) Maastricht University Secretariaat DEC/UNS 50/box 48 Postbus 616 6200 MD Maastricht.

## Author Contributions

AK and TB co-designed the study, performed the experiments, collected and analysed the data, and wrote the manuscript. PL initiated the project. PL, JT, LD, and TB contribute to the writing of the funding grants. LD and JT contributed to the design and the approval of the *in vivo* experiments. All authors contributed to data interpretation, discussions and revised the manuscript.

## Conflict of Interest

PL reports, within and outside the submitted work, grants/sponsored research agreements from Varian medical, Oncoradiomics, ptTheragnostic/DNAmito, Health Innovation Ventures. He received an advisor/presenter fee and/or reimbursement of travel costs/external grant writing fee and/or in kind manpower contribution from Oncoradiomics, BHV, Merck, Varian, Elekta, ptTheragnostic and Convert pharmaceuticals. PL has shares in the company Oncoradiomics, Convert pharmaceuticals, MedC2 and LivingMed Biotech, he is co-inventor of two issued patents with royalties on radiomics (PCT/NL2014/050248, PCT/NL2014/050728) licensed to Oncoradiomics and one issue patent on mtDNA (PCT/EP2014/059089) licensed to ptTheragnostic/DNAmito, three non-patented invention (softwares) licensed to ptTheragnostic/DNAmito, Oncoradiomics and Health Innovation Ventures and three non-issues, non-licensed patents on Deep Learning-Radiomics and LSRT (N2024482, N2024889, N2024889). AK is employed by Exomnis Biotech B.V. Exomnis Biotech did not provide funding and was not involved in the design, execution, analysis, interpretation or publication of the study. The remaining authors declare that the research was conducted in the absence of any commercial or financial relationships that could be construed as a potential conflict of interest. Part of the work reported in this publication is the subject of a pending patent.

## Supplementary Material

Supplementary Table 1 Plasmids and strains used in the study.

Supplementary Table 2 List of oligonucleotides used in the study (restriction enzyme recognition sites underlined).

Supplementary Table 3 Promoter-signal sequence module for pATB1C-XmIL2F variants.

Supplementary Table 4 Colony forming unit counts on tissue samples following the *in vivo* colonisation study.
