# Supplementary material for: Efficient Secretion of Murine IL-2 From an Attenuated Strain of Clostridium sporogenes, a Novel Delivery Vehicle for Cancer Immunotherapy
Source: Front Microbiol. 2021 Jun 8;12:669488. doi: 10.3389/fmicb.2021.669488 (PMC8217651; doi:10.3389/fmicb.2021.669488)
Supplement: Supplementary Table 1 — Plasmids and strains used in the study. [file Table_1.docx]

# SUPPLEMENTARY MATERIAL

**PYT media recipe, per L:** Vegetable peptone 20 g (Sigma, 18332), Veggie yeast extract
15 g (Sigma/Novagen, 71279), Sodium thioglycolate 1 g (Sigma, T0632), Bacteriological agar 1.5 % (Oxoid/Thermo Scientific, LP0011B), pH 7.4

**Spore purification method**

Impure spore suspensions were centrifuged (10,000 *x g,* 10 min, 4 °C) and resuspended in
10 ml of 20 % (v/v) cold Histodenz™ solution (Sigma, D2158). This suspension was layered on top of 30 ml 50 % (w/v) Histodenz™ solution (4 °C) and centrifuged (15,000 x g, 60 min,
4 °C). Subsequently, the Histodenz™ solutions were removed using a pipette, taking care not to disturb the pelleted spores at the bottom. Spore pellets were resuspended in 1 ml cold PBS with 0.05 % Tween 80 (ST80) and centrifuged. Spore prep was washed a further two times with the PBS/0.05 % ST80 solution. Final spore suspensions were resuspended in 5 ml dH_2_O with 0.05 % ST80 and maintained at 4 °C for routine use or at –20 °C for long-term storage. The concentration and purity of spore suspensions was determined by microscopic observation and haemocytometer count.

**Detailed MTT assay**

CTLL-2 indicator cell line (Mouse C57b1/6 T cell) was ordered from ECACC cell culture collection. They were revived in RPMI medium with relevant supplementation (10% FBS and 10% T-STIM). After 10-14 days of undisturbed growth, cell line was maintained and incubated under 5% CO; 37°C. Finally, cell sample have been stained with 0.4% trypan blue to evaluate viability. When satisfactory viability was achieved, cell culture was harvested by centrifugation (200 x g) and washed three times in RPMI-1640. The cell viability was checked using 0.4% trypan blue staining and cells were counted in the haemocytometric chamber under the microscope. Finally, the cells were re-suspended in Culture Medium at the density of 3x105 cells/ml.

100 μl of Culture medium (RPMI-1640 + 10% FBS) was added to each well in 96-well plate; Samples and standards were diluted by performing 2-fold serial dilution from row 2 to 12, (row 1 empty): **Standard:** Recombinant mouse Interleukin-2 was used (#IL031 Sigma). Working concentration was prepared (40 ng/ml) and the assay range included 10 ng/ml in row 2 to 0.0097 ng/ml in row 12. For most in vitro applications, IL-2 exerts its biological activity in the concentration range of 0.1 to 1.0 ng/ml (rows 5-9); **Samples:** *C. sporogenes-*NT plasmid based IL2: (p5, p6, p8, p9), *C. sporogenes* NT and pATB controls were streaked and inoculated in 10 ml fresh PYG media. After 7-hour growth, 1ml samples were obtained from all strains. Samples were spun down, and supernatants were filter sterilised using 0.22 μm filter and syringe. 100 μl of washed CTLL-2 cells (3x10^5^ cells/ml) was added to each well. Cells were incubated for 48 hours at 5% CO_2_; 37°C in a humidified incubator. 10 μl of 5 mg/ml MTT solution was added to each well and plate was incubated for 4 hours. 50 μl of Lysing Solution (20% SDS in 50% DMF) was added to the plate and incubated overnight. Results were recorded by spectrophotometric measurement at 570 nm

**TABLE S1: Plasmids and strains used in the study**

| **Plasmids** | **Relevant characteristics** | **Source** |
| --- | --- | --- |
| pMTL82121 | *E*. *coli*-clostridia shuttle vector | Heap *at al.*([Heap et al., 2009](#_ENREF_16)) |
| pPME-101-g1 | CRISPR-Cas9 vector, targeting SLS operon with guide number 1 | This study |
| pPME-101-g2 | CRISPR-Cas9 vector, targeting SLS operon with guide number 2 | This study |
| pATB1C-XmIL2F | *E*. *coli*-clostridia shuttle vector with FLAG-tagged and codon optimised murine IL-2. “X” represents the promoter-signal sequence module: p5, P*fdx*-eglA; p6, P*fdx*-nprM3; p8, P*ptb*-eglA; p9, P*ptb*:nprM3 | This study |
| **Strains** | **Description** | **Source** |
| *E. coli* 10-beta | Expression/ plasmid storage strain (Δ(ara-leu) 7697 araD139 fhuA ΔlacX74 galK16 galE15 e14- ϕ80dlacZΔM15 recA1 relA1 endA1 nupG rpsL (StrR) rph spoT1 Δ(mrr-hsdRMS-mcrBC)) | NEB^®^ C3019 |
| *E. coli* S17-1 | Conjugative donor strain (recA pro hsdR RP4­2­Tc::Mu­Km::Tn7 integrated into the chromosome) | ATCC 47055™ |
| *C. sporogenes-*WT/ CspWT | *Clostridium sporogenes* NCIMB 10696 wild type strain | NCIMB culture collection (UK) |
| *C. butyricum /*Cbut-M588 | *Clostridium butyricum* MIYAIRI 588 wild type strain | Miyarisan Pharmaceutical Co. LTD. (Japan) |
| *C. sporogenes-*NT/ CspNT | *Clostridium sporogene*Δ*SLS::BM1 -* SLS operon KO strain with inserted BM1 bookmark | This study |
| CspNTΔ*spo0A* | *Clostridium sporogenes-*NTΔ*spo0A* - SLS operon and spo0A KO strain for the control of heat treatment | Precision Medicine (UM, the Netherlands) |
| *C. sporogenes-*NT-XmIL2F (p5, p6, p8, p9) | SLS operon KO strain secreting plasmid-based, FLAG-tagged murine IL2. “X” represents the promoter-signal sequence module: p5, P*fdx*-eglA; p6, P*fdx*-nprM3; p8, P*ptb*-eglA; p9, P*ptb*-nprM3 | This study |
| *C. sporogenes*-NT-pMTL82121 | *C. sporogenes*-NT (SLS operon KO strain) with conjugated pMTL82121 plasmid | This study |
| *S. pyogenes /* Spy-20565 | *Streptococcus pyogenes* DSM20565: group A wild type strain | DSMZ Culture collection (Germany) |
| *C. novyi*-NT | Attenuated strain of *Clostridium novyi* | Provided by Bert Vogelstein (Department of Oncology, Johns Hopkins University School of Medicine) |

**Table S2: List of oligonucleotides used in the study** (restriction enzyme recognition sites underlined).

| **Oligonucleotide name** | **Sequence (5’-3’)** | **Description** |
| --- | --- | --- |
| T1Uni-NotI-F | TATAGCGGCCGCCTGCCAATAGATAAAATAAAGTCTGCC | Amplification of promoter-signal sequence module for mIL-2 |
| eglA-BsaI-R | CCGGTCTCAGAGCAGCTTCAGCTTTATAAGTATTTGTTCCTAATACTG |  |
| nprM3-BsaI-R | CCGGTCTCAGAGCAGCATAAACTGCAGAAACAGTAGAAAGAGTG |  |
| mIL2F-BsaI-F | CCGGTCTCAGCTCCAACTTCTAGTTCAACTTCAAGTTC | Amplification of mIL-2F |
| mIL2F-XhoI-R | TATACTCGAGTTATTATTTATCATCATCATCTTTATAATCTTGAGG |  |
| M13-R | CAGGAAACAGCTATGAC | Screening primer for pATB1C-XmIL2F |
| Cas9-F | TATAGGTCTCTTAGAATGGATAAGAAATACTCAATAGGC | Amplification of Cas9 from  *S. pyogenes* M1 |
| Cas9-R | ATATGCGGCCGCTCAGTCACCTCCTAGCTGAC | Amplification of Cas9 from  *S. pyogenes* M1 |
| Pthl-F | TATATCTAGATTTTTAACAAAATATATTGATAAAAATAATAATAG | Amplification of Pthl from  *C. acetobutylicum* ATCC 824 |
| Pthl-R | TATAGGTCTCTTCTAACTAACCTCCTAAATTTTGATACG | Amplification of Pthl from  *C. acetobutylicum* ATCC 824 |
| ParaE-F | TATATCTAGATTTATATTTAGTCCCTTGCC | Amplification of ParaE from  *C. acetobutylicum* ATCC 824 |
| ParaE-R | TATAGTCGACGAAAACTCCTCCTTAAG | Amplification of ParaE from  *C. acetobutylicum* ATCC 824 |
| LHA-F | TATAGGTCTCGGGAAAATAAAGAAGGACAAGTATTGCTAAATTCTTATGATGTAG | Amplification of left and right homology arm to generate the editing template to delete SLS operon from CspWT |
| LHA-R | TATAGGTCTCGTCTCTGTTAACCCTCAAATTTATTATACCTAATAATCCTTTTTAATTATTTTCATGTACAC |  |
| RHA-F | TATAGGTCTCGGAGATTTACACAGAGGCCCTTAGTATAGTAATTTTATTTGCAGTAAGTTTCTTTATTATAGG |  |
| RHA-R | TATAGGTCTCGTGGCCCAAAGTTCACTAATTTTAACTTTAACAAGAAACCTG |  |
| sgRNA-UNI-R | TATAGACGTCATAAAAATAAGAAGCCTGCAAATGCAGGCTTCTTATTTTTATAAAAAAAGCACCGACTCGGTGCCACTTTTTCAAGTTG | Guide RNA 3’and terminator, for construction of complete guide RNAs in primer dimer reaction. |
| SLSg1-SalI-F | TATAGTCGACGAGTTAATCCATCTGCAGGAGTTTTAGAGCTAGAAATAGCAAGTTAAAATAAGGCTAGTCCGTTATCAACTTGAAAAAGTGGCACCGAGTCGGTGCTTTTTTTATAAAAA | Creation of guide number 1 retargeting sequence in combination with sgRNA-UNI-R |
| SLSg2-SalI-F | TATAGTCGACATATACTTTATTATACCCAAGTTTTAGAGCTAGAAATAGCAAGTTAAAATAAGGCTAGTCCGTTATCAACTTGAAAAAGTGGCACCGAGTCGGTGCTTTTTTTATAAAAA | Creation of guide number 2 retargeting sequence in combination with sgRNA-UNI-R |
| SLS-Flank-F | TAGCATCAGGAGGAACGAAGATAAAGGC | Colony PCR chromosomal screening primers on putative heamolysin KO (CspNT). |
| SLS-Flank-R | CCATAAATCTCTCAATATGTCAAAGCCATCAAGTCC |  |
| SLS-Junc-R | ACCGCCACCTACACTAACACTAACGC |  |
| BM1-R | AAGGGCCTCTGTGTAAATCTCTGTTAACCC |  |

**Table S3: Promoter-signal sequence module for pATB1C-XmIL2F variants**

| **Variant** | **Synthesized sequence** |
| --- | --- |
| p5 | gcggccgc**GTGTAGTAGCCTGCGAAATAAGTAAGGAAAAAAAAGAAGTAAGTGTTATATATGATGATTATTTTGTAGATGTAGATAGGATAATAGAATCCATAGAAAATATAGGTTATACAGTTATATAAAAATTACTTTAAAAATTAATAAAAACATGGTAAAATATAAATCGTATAAAGTTGTGTAATTTTTAAGGAGGTGTGTTACAT***atgttttcaaaaatcaaaaaaattaatttttttaaaaaaacattttcttttttaattgctgttgtaatgatgttgtttacagtattaggaacaaatacttataaagctgaagct*gctctgagacc |
| p6 | gcggccgc**GTGTAGTAGCCTGCGAAATAAGTAAGGAAAAAAAAGAAGTAAGTGTTATATATGATGATTATTTTGTAGATGTAGATAGGATAATAGAATCCATAGAAAATATAGGTTATACAGTTATATAAAAATTACTTTAAAAATTAATAAAAACATGGTAAAATATAAATCGTATAAAGTTGTGTAATTTTTAAGGAGGTGTGTTACAT***atgaaaagtaaaaaattattagctacagtgctaagtgctgtaatcactctttctactgtttctgcagtttatgct*gctctgagacc |
| p8 | gcggccgc**TAAGTCAGCAGAAAGTATAATGAGAAAATATAAAATATAAATAATTTTCTAAAAAACTTAACTTCATGTGAAAAGTTTGTTAAAATATAAATGAGCACGTTAATCATTTAACATAGATAATTAAATAGTAAAAGGGAGTGTACGACCAA***tgttttcaaaaatcaaaaaaattaatttttttaaaaaaacattttcttttttaattgctgttgtaatgatgttgtttacagtattaggaacaaatacttataaagctgaagct*gctctgagacc |
| p9 | gcggccgc**TAAGTCAGCAGAAAGTATAATGAGAAAATATAAAATATAAATAATTTTCTAAAAAACTTAACTTCATGTGAAAAGTTTGTTAAAATATAAATGAGCACGTTAATCATTTAACATAGATAATTAAATAGTAAAAGGGAGTGTACGACCA***atgaaaagtaaaaaattattagctacagtgctaagtgctgtaatcactctttctactgtttctgcagtttatgct*gctctgagacc |

**Legend:**

- Underlined: restriction enzyme recognition site;
- in **bold blue**: P*fdx* ferredoxin promoter (associated with the protein coding gene CLSPO_c0087 in *C. sporogenes* NCIMB 10696);
- in **bold yellow**: P*ptb* promoter (associated with the protein coding gene Ca_30776 in *C. acetobutylicum* ATCC 824);
- in *italicized purple*: Endoglucanase EglA precursor sequence (associated with the protein coding gene CLSA_c09900 in *C. saccharobutylicum* DSM 13864);
- in *italicized red*: metalloprotease NprM3 precursor sequence (associated with the protein coding gene CLSPO_c14710 in *C. sporogenes* NCIMB 10696.

**Codon optimised Flag-tagged murine IL2**

ggtctcagctccaacttctagttcaacttcaagttctacagctgaagcacaacaacagcaacaacagcaacaacaacagcaacaacatcttgaacaactattaatggatctacaagaacttctatctagaatggaaaattatagaaaccttaaactaccaagaatgctaacatttaaattttatttaccaaaacaagcaacagaattaaaagatcttcagtgtctcgaagatgaacttggtcctctacgtcatgttctagatttaactcaaagtaaaagttttcaattggaagatgcagaaaattttataagtaatattagagtaactgttgtaaaactaaagggatctgataacacttttgaatgtcaattcgatgatgaatcagctactgttgtagattttctaagaaggtggatagcattctgtcaaagtatcatatctacaagtcctcaaGATTATAAAGATGATGATGATAAAtaataactcgag

Underlined: restriction enzyme recognition site;
IN CAPITAL: FLAG-Tag sequence

**Table S4: Colony forming unit counts on tissue samples following the *in vivo* colonization study**

|  |  | Tumour | | Spleen | | Lymph Nodes | |
| --- | --- | --- | --- | --- | --- | --- | --- |
| Animal | Group | Spore | Vegetative | Spore | Vegetative | Spore | Vegetative |
| S1 | Spores | 2.8E+06 | 2.2E+07 | 3.7E+02 | 4.3E+02 | 1.0E+02 | 1.4E+03 |
| S2 | Spores | 3.5E+06 | 2.0E+07 | 9.0E+02 | 1.5E+03 | 2.0E+02 | 1.3E+03 |
| S3 | Spores | 5.3E+06 | 7.0E+07 | 5.2E+02 | 7.0E+02 | 1.3E+01 | 1.2E+02 |
| S4 | Spores | 9.9E+05 | 4.5E+06 | 7.0E+02 | 4.2E+02 | 3.3E+01 | 6.3E+02 |
| S5 | Spores | 8.1E+05 | 2.9E+06 | 3.7E+02 | 5.67E+02 | 3.3E+01 | 2.67E+02 |
| S6 | Spores | 2.6E+06 | 7.3E+06 | 5.2E+02 | 4.17E+02 | 5.7E+02 | 5.33E+02 |
| S7 | Spores | 1.4E+05 | 1.0E+06 | 7.0E+02 | 1.83E+02 | 8.3E+01 | 6.67E+02 |
| S8 | Spores | 1.1E+05 | 2.2E+06 | 1.3E+02 | 3.20E+02 | 1.0E+02 | 4.00E+02 |
| C1 | PBS | 0.0E+00 | 0.0E+00 | 0.0E+00 | 0.0E+00 | 0.0E+00 | 0.0E+00 |
| C2 | PBS | 0.0E+00 | 0.0E+00 | 0.0E+00 | 0.0E+00 | 0.0E+00 | 0.0E+00 |
